# Error Analysis of the Combined-Scan High-Speed Atomic Force Microscopy

**DOI:** 10.3390/s21186139

**Published:** 2021-09-13

**Authors:** Lu Liu, Ming Kong, Sen Wu, Xinke Xu, Daodang Wang

**Affiliations:** 1 College of Metrology and Measurement Engineering, China Jiliang University, Hangzhou 310018, China; mkong@cjlu.edu.cn (M.K.); xuxinke-123@outlook.com (X.X.); wangdaodang@cjlu.edu.cn (D.W.); 2 State Key Laboratory of Precision Measurement Technology and Instruments, Tianjin University, Tianjin 300072, China; senwu@tju.edu.cn

**Keywords:** atomic force microscopy, combined-scan, error analysis, Piezo, Zemax

## Abstract

A combined tip-sample scanning architecture can improve the imaging speed of atomic force microscopy (AFM). However, the nonorthogonality between the three scanners and the nonideal response of each scanner cause measurement errors. In this article, the authors systematically analyze the influence of the installation and response errors of the combined scanning architecture. The experimental results show that when the probe in the homemade high-speed AFM moves with the Z-scanner, the spot position on the four-quadrant detector changes, thus introducing measurement error. Comparing the experimental results with the numerical and theoretical results shows that the undesired motion of the Z-scanner introduces a large error. The authors believe that this significant error occurs because the piezoelectric actuator not only stretches along the polarization direction but also swings under nonuniform multifield coupling. This article proposes a direction for further optimizing the instrument and provides design ideas for similar high-speed atomic force microscopes.

## 1. Introduction

Atomic force microscopy (AFM) can be used to measure the topography and physicochemical properties of materials at the nanometer scale and to manipulate and fabricate a variety of functional nanostructures; this technique has become one of the most important tools in nanotechnology [1,2,3,4,5,6,7]. AFM, in general, includes five major components: the cantilever probe, the detection system, the tip-sample motion system, the feedback controller, and the image processing and display system [8]. To detect the bending of the cantilever, various methods, such as optical interferometric [9,10], optical lever [1,8,11], and capacitance sensing [12] methods, are employed. Among them, the most commonly used detection method is the optical lever detection method. To ensure measurement accuracy of the AFM, the laser must always track the cantilever probe, and the focused spot on the cantilever must be in the same position during measurements. In sample-scan AFM, both the cantilever probe and the laser spot remain unchanged. There are no laser tracking errors, which is the primary error of the AFM. In tip-scan and combined-scan AFM, the cantilever probe moves with the scanners, and there is mutual movement between the cantilever and the incident laser. Thus, the laser tracking error must be considered in the tip-scan and combined-scan AFM designing [13,14,15]. One of the causes of the laser tracking error is the nonorthogonality of the three scanners. This reflects the installation error and the movement error of each scanner.

Piezoelectric actuators are widely used in ultra-precision positioning, measurement, and machining systems due to their rapid dynamic performance, ultra-high resolution, and simple miniaturization [16,17]. In the AFM, both the tip oscillation and the position of the tip relative to the sample surface are achieved using piezoelectric actuators. A piezoelectric tube that enables motion in the *x*-, *y*-, and *z*-directions is the scanner of choice in most instruments [18,19]. However, there is some coupling between the lateral and vertical displacements of the piezoelectric tube scanner. Therefore, a scanner structure of a stack piezoelectric actuator or piezoelectric sheet combined with a flexure hinge is now widely studied and applied [20,21,22]. In this structure, three piezoelectric actuators are needed to realize scanning motions in the *x*-, *y*-, and *z*-directions. The separation structure must ensure the orthogonality of the three movement directions. On one hand, the orthogonality of each actuator must be ensured during installation, which can be achieved by high-precision machining. On the other hand, the movement direction of each actuator must be straight.

According to the piezoelectric equation [23,24], the motion response of the actuator is related not only to the excitation voltage but also to the external environment, which affects the piezoelectric constant, elastic matrix, and so on [25,26,27]. The nonlinearity between the mechanical deformation and the voltage can be compensated by closed-loop control. However, the influence of multifield coupling on the actuator motion characteristics is more complex. Under multifield coupling, especially nonuniform multifield coupling, the moving direction of the actuator may not be parallel to the polarization direction. This phenomenon greatly influences the measurement results and cannot easily be compensated.

In this article, the authors took a homemade combined-scan high-speed AFM as an example. They discuss the influence on the laser tracking errors of the installation and response error of the XYZ scanner [28,29]. First, this article introduces the specific structure of the high-speed AFM. Then, the installation and response state of the XYZ scanner is analyzed and tested by using a combination of numerical simulation, theoretical derivation, and experimental studies. The results show the main error sources of the instrument, which can provide a theoretical and empirical basis for further optimization of the instrument.

## 2. Modelling of Scanner Installation Errors

### 2.1. Homemade High-Speed Atomic Force Microscopy

**Figure 1:**
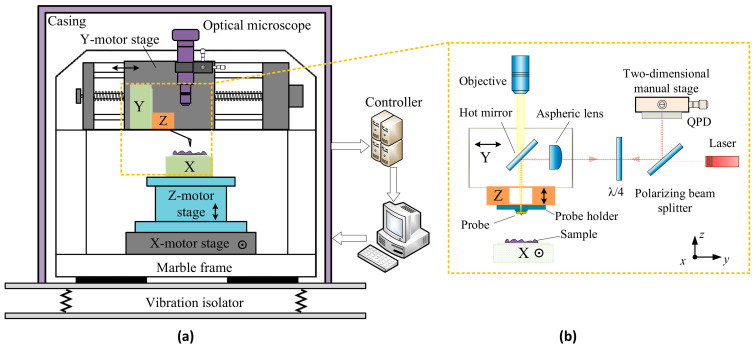
Schematic layout of the novel HS-AFM system. (**a**) The mechanical structure of the novel HS-AFM system; (**b**) schematic diagram of the core structure of the HS-AFM.

Figure 1a shows the homemade combined-scan high-speed atomic force microscopy (HS-AFM) [28]. The developed apparatus is mainly constructed of a marble frame and an air-supporting vibration isolator. Three precision motion stages are installed orthogonally for large-area sample positioning and the tip-sample approach. An optical microscope and the AFM head, including a Y-scanner and Z-scanner, are mounted on the Y-motor stage. The X-scanner is mounted on the Z-motor stage, which is fixed on the X-motor stage. Figure 1b shows the schematic diagram of the core structure of the HS-AFM. The X-scanner is separated from the AFM head and is used to drive the sample scan along the *x*-axis. The Z-scanner is used to oscillate a cantilever probe and implement feedback motion along the *z*-axis. The Z-scanner, hot mirror, and aspheric lens are mounted on the Y-scanner and scanned along the *y*-axis. An optical beam detector (OBD) is used to detect the cantilever deformation. To maintain a sufficiently small laser spot focused on the cantilever probe, an aspheric lens (*f* = 20 mm, the working distance (WD) = 15.7 mm) with a numerical aperture (NA) of 0.543 and a design wavelength of 780 nm is used as a focusing lens. The diverging laser beam reflected from the cantilever is reflected again by the hot mirror and then collimated by the aspheric lens. After passing back through the quarter-wave plate, the laser beam returning to the polarizing beam-splitter (PBS) is fully reflected by the splitter face and reaches the quadrant photodiode detector (QPD). The QPD is mounted on a two-dimensional manual stage, which helps with the fine adjustment of the initial position of the laser spot on the QPD.

### 2.2. Theoretical Analysis of the Installation Errors of the Scanners

In the process of instrument construction, the motion of the XYZ scanners does not cause measurement errors if the mutual position relationship and movement of each element are consistent with the design requirements. However, due to the design, manufacturing, and assembly errors, the matching relationship between the components cannot be entirely consistent with the theoretical setting values. Therefore, a theoretical analysis of the installation errors is described in this section.

#### 2.2.1. X-Scanner

As shown in Figure 1, the X-scanner is separated from the AFM head, and its motion does not affect the OBD, which is used to detect the cantilever deformation. However, if the moving direction of the X-scanner is not orthogonal to that of the Y-scanner or the Z-scanner, the obtained sample topography will be distorted.

**Figure 2:**
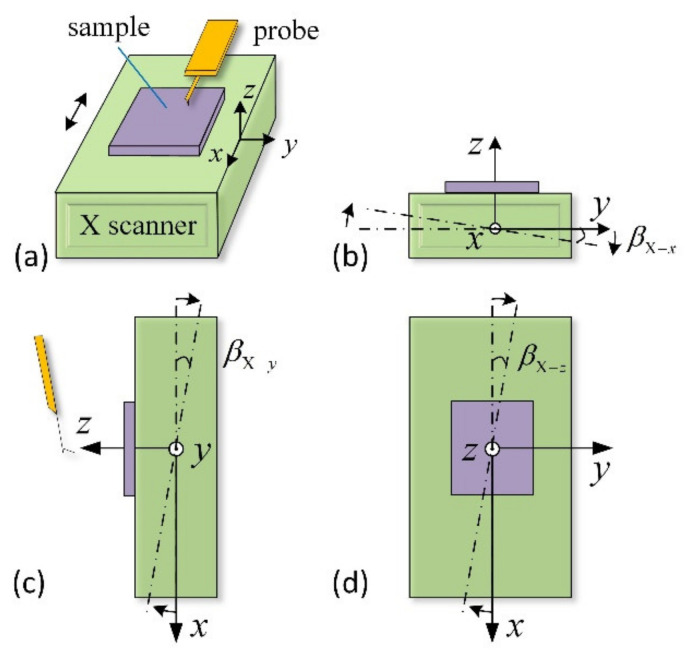
Installation error of the X-scanner. (**a**) Normal installation diagrams. Installation diagrams of an X-scanner installed obliquely around the *x*-axis (**b**), around the *y*-axis (**c**), and around the *z*-axis (**d**).

Figure 2 shows three typical cases of an obliquely installed X-scanner. If the X-scanner is installed obliquely, as shown in Figure 2b,c, the sample stage is not orthogonal to the *z*-axis, resulting in an obliquity sample surface. However, this obliquity phenomenon can be easily corrected by the least square method. When the X-scanner is inclined around the *z*-axis by angle $\beta_{X-z}$ (as shown in Figure 2d), the surface geometry of the measured sample topography is distorted, and the distortion angle is consistent with the angle ($\beta_{X-z}$). This phenomenon can be detected and corrected by imaging the standard calibration grid.

#### 2.2.2. Y-Scanner

In the homemade high-speed AFM, the Z-scanner, the hot mirror, and the aspheric lens are mounted on the Y-scanner and move with it. The cantilever probe is fixed on the Z-scanner by the probe holder. The movement of the Y-scanner and the Z-scanner, which are integrated in the AFM head, may cause laser tracking errors.

In this section, the optical axis of the aspheric lens is assumed to be parallel to the moving direction of the Y-scanner. Thus, if the installation of the Y-scanner is ideal and its moving direction is parallel to the laser incidence direction, the focused spot can always remain at the same point on the cantilever during scanning in any arbitrary range along the *y*-axis. The movement of the Y-scanner does not change the spot position on the QPD, that is, it does not introduce measurement error. However, if the moving direction of the Y-scanner is not parallel to the direction of laser incidence, the motion of the Y-scanner leads to measurement error.

**Figure 3:**
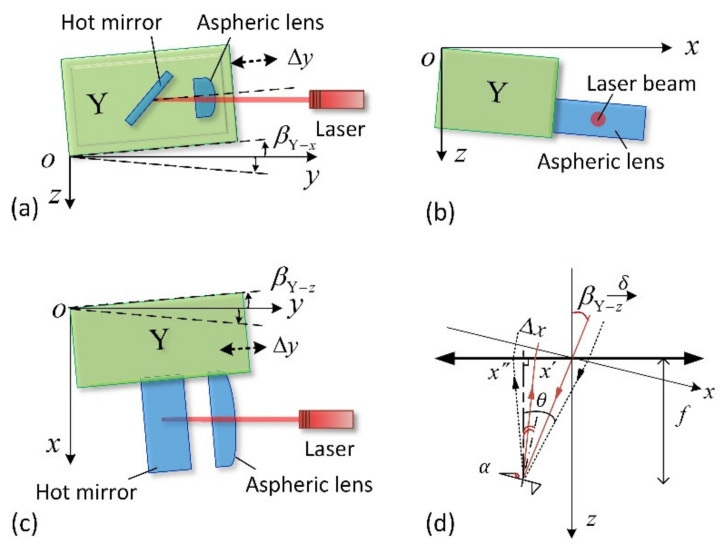
Typical schematic diagram of an obliquely installed Y-scanner. Installation diagrams of the Y-scanner installed obliquely around the *x*-axis (**a**), around the *y*-axis (**b**). Installation diagrams (**c**) and the simplified optical path (**d**) of the Y-scanner installed obliquely around the *z*-axis.

Figure 3 shows three typical cases of an obliquely installed Y-scanner. Suppose the Y-scanner is installed obliquely, as shown in Figure 3a. In that case, its movement changes the relative position between the aspheric lens and the incident laser beam in the *z*-direction, leading to the laser spot’s movement on the QPD along the *y*-axis. When the Y-scanner is installed obliquely in the *xoz*-plane, as shown in Figure 3b, its moving direction is still parallel to the laser incident direction. Thus, the movement of the Y-scanner does not change the laser spot position on the QPD, that is, the Y-scanner does not affect the measurement results.

Figure 3c shows another tilted installation of the Y-scanner. In this case, the optical axis of the aspheric lens is parallel to the moving direction of the Y-scanner, and the laser is tilted into the aspheric lens. With the movement of the Y-scanner, the incident laser position on the principal plane of the aspheric lens moves along the *x*-axis. Figure 3d shows the simplified schematic diagram of the OBD system. Suppose the installation inclination of the Y-scanner is $\beta_{Y-z}$, and the elongation in the direction of the dotted arrow is Δy. In that case, the variation in the spot position on the principal plane of the aspheric lens can be described as $\delta_{Y-z}=\Delta y\cdot \tan\beta_{Y-z}$, and the displacement of the laser spot on the QPD $\Delta x_{Y-z}$ can be described as:

(1) $$\left\{\begin{array}{l} \Delta x_{Y-z}\approx \gamma \cdot f\cdot [\tan(2\alpha +\theta )-\tan(2\alpha +\beta_{Y-z})]\cdot \cos\beta_{Y-z}\\ \tan\theta =(f+\Delta y)\cdot \tan\beta_{Y-z}/f \end{array}\right.$$

where *f* is the focal length of the aspheric lens, *α* is the initial inclination angle of the cantilever, and $\beta_{Y-z}$ is the inclination angle of the Y-scanner shown in Figure 3c, in which $\beta_{Y-z}$ > 0 when installed clockwise and $\beta_{Y-z}$ < 0 when installed anticlockwise. *γ* is the correlation coefficient, which is inversely proportional to the radius of the incident laser beam.

#### 2.2.3. Z-Scanner

In the homemade high-speed AFM, the Z-scanner drives the cantilever to scan along the *z*-axis and oscillate, but the aspheric lens position remains unchanged. Therefore, the expansion of the Z-scanner, especially the expansion in an extensive range, changes the spot position on the QPD, thus introducing measurement error.

**Figure 4:**
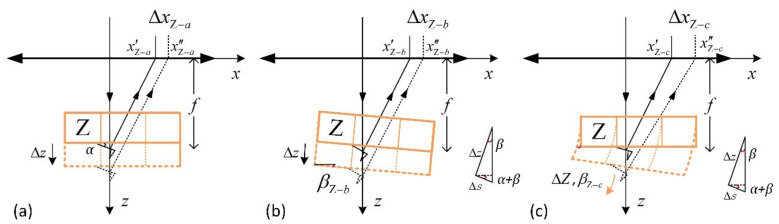
Three types of installation and extension of the Z-scanner. Optical diagram under ideal conditions (**a**), obliquely installation (**b**) and with distorted expansion trajectory (**c**).

Figure 4a shows the optical diagram under ideal conditions. As the Z-scanner moves, the reflected laser spot on the principal plane of the aspheric lens (approximately on the QPD) moves accordingly. In this case, the reflected laser spot displacement on the principal plane of the aspheric lens caused by the expansion of the Z-scanner can be expressed as:

(2) $$\Delta x_{Z-a}=\gamma \cdot \Delta z\cdot \tan2\alpha$$

where Δz is the expansion of the Z-scanner and *α* is the initial inclination angle of the cantilever.

Suppose the Z-scanner is installed obliquely, as shown in Figure 4b. In that case, its movement results in the displacement of the focused spot position on the cantilever and the displacement of the laser spot on the QPD, and these displacements can be expressed as:

(3) $$\left\{\begin{array}{l} \Delta s_{Z-b}=\Delta z\cdot \sin\beta_{Z-b}/\cos(\alpha +\beta_{Z-b})\\ \Delta x_{Z-b}\approx \gamma \cdot \Delta z\cdot (1+\tan\beta_{Z-b}\tan(\alpha +\beta_{Z-b}))\cdot \tan(2\alpha +2\beta_{Z-b})\cdot \cos\beta_{Z-b} \end{array}\right.$$

where $\Delta s_{Z-b}$ is the displacement of the focused spot on the cantilever and $\Delta x_{Z-b}$ is the spot displacement on the QPD, which are caused by the obliquely installed Z-scanner shown in Figure 4b. $\beta_{Z-b}$ is the inclination angle of the Z-scanner, in which $\beta_{Z-b}$ > 0 when installed clockwise and $\beta_{Z-b}$ < 0 when installed anticlockwise.

In addition, a ring-shaped piezoelectric actuator is used as the Z-scanner in the designed system. Due to the influence of the external nonuniform force field and thermal field during installation and use, the expansion and contraction trajectory of the piezoelectric actuator may be distorted, as shown in Figure 4c, rather than an ideal straight line. In this case, the focused spot position on the cantilever and the cantilever’s tilt angle will change. The resulting spot displacements on the cantilever ($\Delta s_{Z-c}$) and on the QPD ($\Delta x_{Z-c}$) can be described as:

(4) $$\left\{\begin{array}{l} \Delta s_{Z-c}=\Delta z\cdot \sin\beta_{Z-c}/\cos(\alpha +\beta_{Z-c})\\ \Delta x_{Z-c}=\gamma \cdot ((f+\delta )\tan(2\alpha +2\beta_{Z-c})-f\tan(2\alpha ))\\ \delta_{Z-c}=\Delta z\cdot (1+\tan\beta_{Z-c}\tan(\alpha +\beta_{Z-c}))\cdot \cos\beta_{Z-c} \end{array}\right.$$

where Δz is the expansion of the Z-scanner, *α* is the initial inclination angle of the cantilever, $\beta_{Z-c}$ is the inclination angle of the cantilever, *f* is the focal length of the aspheric lens, and $\delta_{Z-b}$ is the displacement of the free end of the cantilever along the *z*-direction shown in Figure 4c.

**Figure 5:**
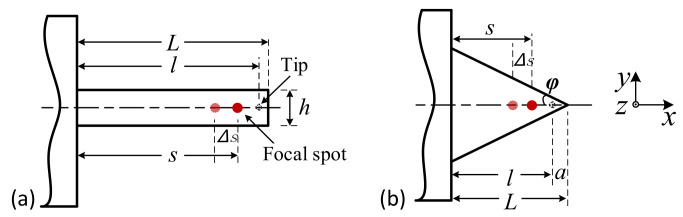
Diagram of focused spot movement on the cantilever. (**a**) Rectangular cantilever; (**b**) triangular cantilever.

The displacement of the focused spot on the cantilever Δs also changes the magnification of the OBD. As shown in Figure 5, the magnifications of the OBD corresponding to a rectangular cantilever (*A_Rec_*) and triangular cantilever (*A_Tri_*) can be described as:

(5) $$\left\{\begin{array}{l} A_{Rec}=\frac{6fs}{l^{3}}(l-\frac{s}{2})\\ A_{Tri}=4f\frac{s+a\ln\frac{L-s}{L}}{l^{2}-2a^{2}\ln\frac{a}{L}-2al} \end{array}\right.$$

If there is a small displacement of the focused spot on the cantilever along the *x*-direction, the magnification of the optical lever changes. Therefore, when the displacement of the focused spot on the cantilever along the *x*-direction is Δs and the displacement of the probe tip along the *z*-direction is Δz, the spot displacement on the QPD (Δx) can be expressed as:

(6) $$\left\{\begin{array}{l} \Delta x_{Rec}\approx \frac{6f\Delta z\Delta s}{l^{3}}(\frac{\Delta s}{2}+l-s)\\ \Delta x_{Tri}\approx 4f\Delta z\frac{\Delta s+a\ln\frac{L-s}{L-s+\Delta s}}{l^{2}-2a^{2}\ln\frac{a}{L}-2al} \end{array}\right.$$

where *f* is the focal length of the aspheric lens, *s* is the distance between the focused spot position and the fixed end of the cantilever, *l* is the distance between the tip and the fixed end of the cantilever, *L* is the length of the cantilever, *a* is the distance between the tip and the free end of the cantilever, and *a* = *L* − *l*.

## 3. Optical Simulation

**Figure 6:**
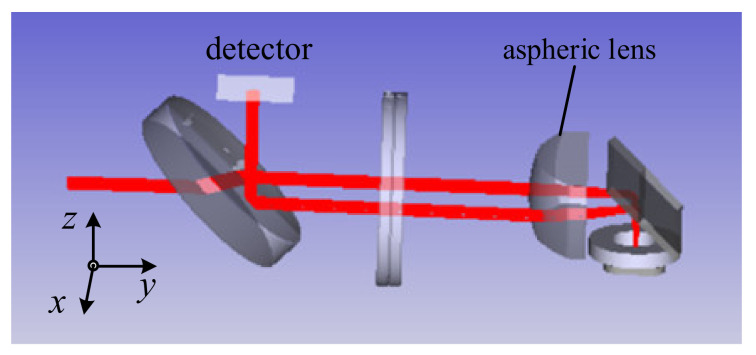
The layout of the Zemax model.

In the system, the scanners and the optical lens may produce an axial error, angular error, etc. These errors will lead to laser tracking errors and decrease the measurement accuracy of the AFM. In this section, we use Zemax and MATLAB to analyze the installation errors of the scanners. Figure 6 shows the simulation model based on Zemax operating in nonsequential mode. First, different parameters were set for the model using MATLAB and transmitted to the Zemax software through dynamic data exchange (DDE) technology. Then, after the ray tracing, the detector matrices were transmitted to MATLAB through DDE technology. Finally, the laser spot positions on the detector were calculated and analyzed.

### 3.1. Y-Scanner

**Figure 7:**
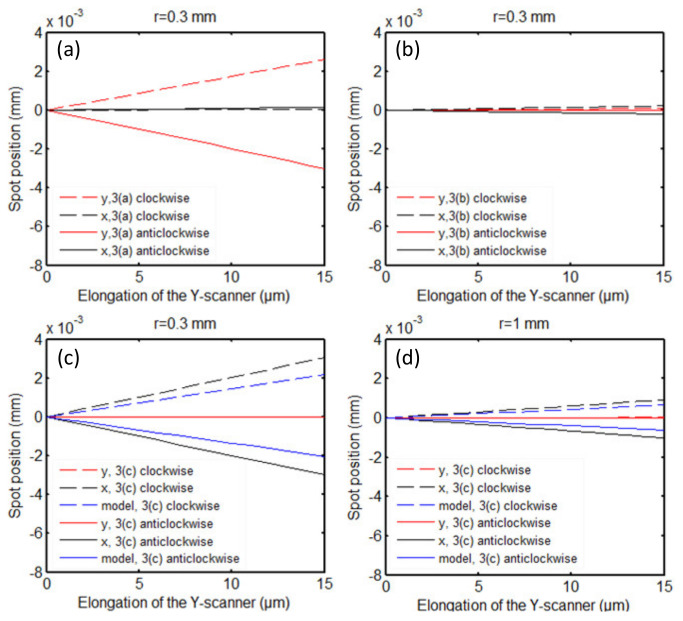
The simulation results of the laser spot movement on the QPD were caused by the extension of the oblique-mounted Y-scanner. (**a**–**c**) The results produced by the Y-scanner movement. When the scanner is mounted obliquely, as shown in Figure 3a–c, the radius of the laser spot on the QPD is 0.3 mm. (**d**) The radius of the laser spot on the QPD is 1 mm. The dotted lines and solid lines indicate that the Y-scanner is mounted obliquely clockwise and anticlockwise, respectively. The blue lines represent the theoretical curve of Formula (1). The tilt angle used in the simulation is 2°.

The simulation analysis was carried out for three typical cases in Figure 3, and the simulation results corresponding to these three scenarios are shown in Figure 7. In the simulation experiment, the scan range of the Y-scanner was 15 μm, the tilted angle of the Y-scanner was 2°, and the radius of the incident laser beam was set to 0.3 mm and 1 mm.

As shown in Figure 3a, the Y-scanner is installed obliquely in the *yoz*-plane. Its scanning movement causes drift of the laser spot on the detector along the *y*-axis, according to the theoretical analysis. The same conclusion is obtained from the simulation, as shown in Figure 7a. With the extension of the tilted Y-scanner, the spot on the QPD moves along the *y*-axis, and the laser spot position along the *x*-axis (the direction of the long axis of the cantilever) remains unchanged (the variation is less than 0.04 μm). When the Y-scanner is obliquely installed clockwise, the spot position increases with scanner extension (dotted red line in Figure 7a), and the maximum displacement is approximately 2.60 μm. When the Y-scanner is installed obliquely anticlockwise, the spot position decreases with the scanner extension (solid red line in Figure 7a), and the maximum displacement is approximately 3.01 μm. In the scenario shown in Figure 3b, the moving direction of the Y-scanner is still the same as the incidence direction of the laser. Therefore, in theory, the spot position does not change. Figure 7b shows that the maximum displacement of the spot is approximately 0.22 μm in this experiment.

According to the theoretical analysis, when the Y-scanner is mounted obliquely, as shown in Figure 3c, its motion strongly impacts the measurement results, as shown by the blue lines in Figure 7c. The black lines (dotted line for clockwise mounting, solid line for anticlockwise mounting) in Figure 7c,d show the consistent simulation results. When the extension of the Y-scanner is 15 μm, the displacement of the laser spot along the *x*-axis on the QPD is 3.06 μm (Figure 7c, r = 0.3) or 0.92 μm (Figure 7d, r = 1). During the testing of the samples, this displacement is superimposed directly on the measured sample topography.

As shown in Figure 7c,d, there is a deviation between the calculated results of the mathematical model and the simulation results. This is because the focused spot movement on the cantilever is not considered in the mathematical model. In addition, we find that the movement of the laser spot on the detector is related to the spot size. Figure 7c,d confirm this conclusion. The smaller the spot is, the larger the displacement of the laser spot on the QPD caused by the oblique-mounted Y-scanner.

### 3.2. Z-Scanner

**Figure 8:**
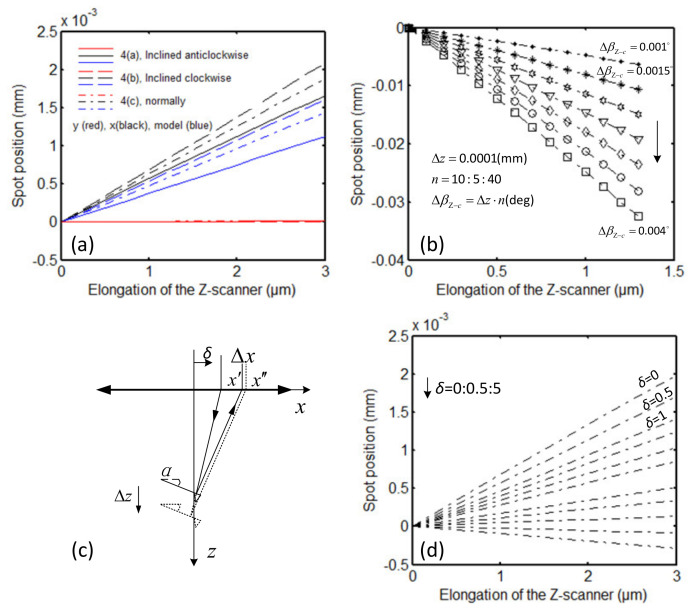
The simulation results of the laser spot movement on the QPD caused by the Z-scanner movement. (**a**) The results for the normal- or oblique-mounted ideal Z-scanner movement, corresponding to Figure 4a,b. (**b**) The results for the Z-scanner movement in nonideal conditions, corresponding to Figure 4c. (**c**,**d**) The incident laser enters the aspheric lens with an offset along the *x*-axis. The blue lines in the figure are the results estimated by the mathematical models.

Figure 8 shows the simulation results of the laser spot movement on the detector caused by the Z-scanner extension. In this simulation, the incident laser beam radius is set to 1 mm.

The laser spot position’s simulation results for the scenario in which the Z-scanner is installed and working, as shown in Figure 4a,b, are shown in Figure 8a. With the movement of the Z-scanner, the spot on the QPD moves along the *x*-axis (black lines) and remains unchanged in the *y*-axis (red lines). The results show that the Z-scanner motion leads to a significant measurement error. The dash-dotted black line indicates the simulation result for the scenario in which the scanner is installed normally. The spot displacement is approximately 1.88 μm when the elongation of the Z-scanner is 3 μm. The dotted and solid lines represent the simulation results when the Z-scanner tilts clockwise (as shown in Figure 4b) and anticlockwise along the cantilever, respectively. The maximum spot displacements are approximately 2.08 μm (clockwise) and 1.66 μm (anticlockwise). Similarly, the difference is that the influence of the spot position change on the cantilever is ignored in the mathematical model.

The simulation results of the laser spot position for the scenario in which the Z-scanner is installed and working, as shown in Figure 4c, are shown in Figure 8b. In this scenario, the cantilever not only moves in the *z*-axis and the *x*-axis, but also tilts. With the extension of the scanner, the inclination angle of the cantilever is assumed to change. In the simulation, with every 0.1 μm of Z-scanner extension, the cantilever moves 0.2 μm along the *x*-axis, and the cantilever angle changes by 0.001°, 0.0015°, …, 0.004° ($\Delta \beta_{Z-c}$). The results show that the spot displacement along the *x*-axis on the QPD is approximately 6.32 μm when the elongation of the Z-scanner is 1.3 μm, and the maximum angle variation of the cantilever is 0.013° (calculated from the line $\Delta \beta_{Z-c}=0.001{}^{\circ}$).

To reduce the measurement error caused by the Z-scanner’s elongation, one method is to change the laser’s incident direction, that is, the angle between the incident laser and the cantilever can be changed, as shown in Figure 8c. Figure 8d shows the simulation results corresponding to different offset *δ*. With increasing offset *δ*, the laser spot displacement Δx decreases. According to the calculation and simulation results, when *δ* is set to 4.25 mm, the incident laser beam is focused vertically on the cantilever, and the displacement Δx caused by the Z-scanner movement is the smallest.

## 4. Experiments and Results

**Figure 9:**
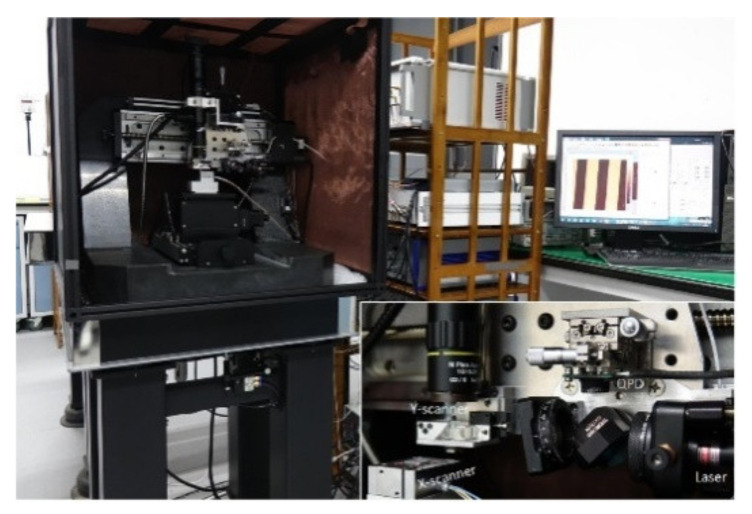
Photo of the novel HS-AFM. Inset: photo of the AFM head.

In this section, the errors caused by the movement of the scanners in the novel HS-AFM are measured. Figure 9 shows a photo of the homemade HS-AFM. In the system, the X-scanner and the Y-scanner are two identical one-dimensional nanopositioning stages (P753.1CD, Physik Instrumente), and the Z-scanner is a ø 12 mm ring piezo actuator (NAC2123, Noliac). All the experimental results were obtained using a FastScan-B cantilever from Bruker. The laser spot radius on the QPD was approximately 1 mm. The measured static sensitivity of this measurement was 18 mV/nm, and the dynamic sensitivity was 9.6 mV/nm. The measured position-voltage conversion rate of the QPD and the corresponding preprocessing circuit was 5.87 mV/μm.

**Figure 10:**
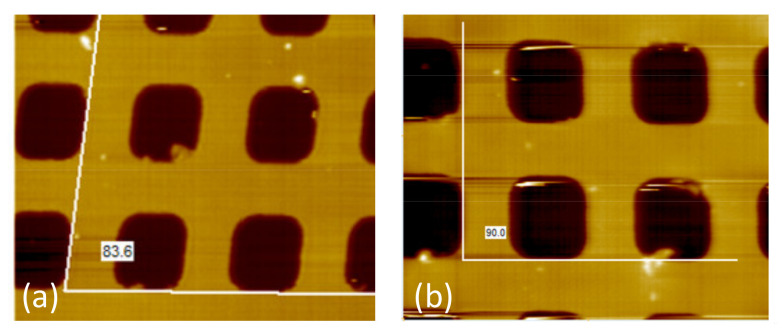
Imaging results of the oblique-mounted (**a**) and normal-mounted (**b**) X-scanners.

As described in Section 2.2.1, the movement of the X-scanner does not change the spot position on the QPD, but it may affect the measured surface profile. Figure 10a shows the distortion of the sample shape caused by the manually obliquely mounted scanner shown in Figure 2d. The angle change of the measured sample shape is the same as the tilt angle of the X-scanner. Simply improving the machining accuracy can reduce this error. Figure 10b shows the imaging result after adjustment of the X-scanner.

**Figure 11:**
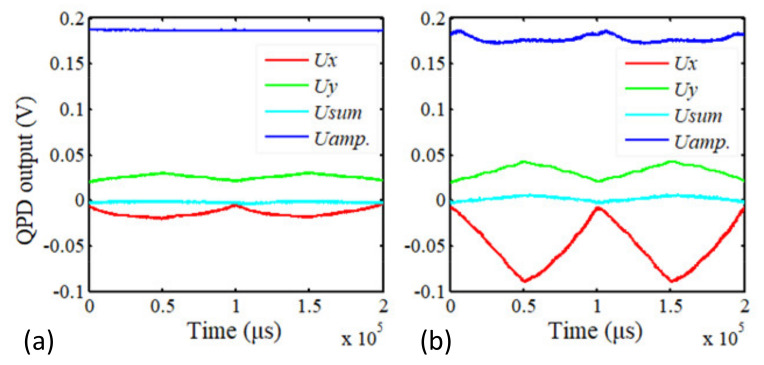
Laser tracking errors caused by the movement of the Y-scanner (**a**) and the Z-scanner (**b**).

Figure 11 shows the output voltages of the QPD caused by scans of the Y-scanner and the Z-scanner. The scan ranges of the Y-scanner and the Z-scanner are 15 μm and 1.25 μm, respectively, and the corresponding driving voltages are 0–10 V and 0–120 V.

Figure 11a shows that the QPD’s output in the *x*-axis ($U_{x}$) is approximately 11 mV, caused by the Y-scanner’s full range scan. The corresponding laser spot displacement on QPD is 1.87 μm, and the corresponding tip displacement is 0.8 nm. Based on the analysis in Section 2.2.2 and Section 3.1, the measurement error is caused by the angle error between the moving direction of the Y-scanner, the optical axis of the aspheric lens, and the laser incidence direction; the total angle is approximately 1.2°. Therefore, a full-range scan with the Y-scanner causes measurement error in contact mode, and the error is about 0.8 nm. The amplitude of the cantilever (blue line) and the total energy of the laser spot ($U_{\mathrm{SUM}}$) are unchanged. This means that the full range scan of the Y-scanner does not affect the measurement in tapping mode.

Figure 11b shows the effect of a scan with the Z-scanner. The change in the cantilever amplitude caused by the Z-scanner movement is 13 mV, and the corresponding tip displacement is estimated to be 1.4 nm. The change in $U_{x}$ is approximately 80 mV, the corresponding spot displacement on QPD is calculated to be 13.63 μm, and the corresponding tip displacement is calculated to be 4.4 nm. If the sample height is 100 nm, the errors introduced by the scan of the Z-scanner in contact mode and tapping mode are 0.36 nm and 0.1 nm, respectively.

Based on the analysis in Section 2.2.3 and Section 3.2, if the Z-scanner is installed normally or obliquely, as shown in Figure 4a,b, the change in $U_{x}=$ 13.64 μm can cause the inclination angle to be nearly 30°, which is impossible. Therefore, the inclination angle of the cantilever must be changed during the Z-scanner extension, and the changed angle is approximately 0.015°, as calculated by Formula (4).

The measurement error caused by the Z-scanner movement can be reduced by moving the position of the incident laser beam along the *x*-axis and keeping the beam vertically focused on the cantilever. In subsequent instrument improvement, it is important to improve the design of the Z-scanner to reduce its distortion, and the probe holder attached to the Z-scanner may need to be optimized.

## 5. Conclusions

In the tip-scan and combined-scan AFMs, the orthogonality of the three scanners and the operation state of the scanner itself will affect the laser tracking result, which affects the measurement accuracy of the AFM system. Taking the homemade combined-scan high-speed AFM as an example, the article introduces and analyses the possible tilt installation situation when installing three scanners and the undesirable response behavior of the piezoelectric ceramic actuator itself. Theoretical and simulation results show that the unideal movement of the Z-scanner used to drive the probe for feedback tracking and resonance response is the main error that affects the measurement accuracy. The height measurement error of 0.1 nm (corresponding to the measured sample height of about 100 nm) in the tapping mode comes from the angle change of the cantilever caused by the extension of the Z-scanner, and the changed angle is approximately 0.015°. Therefore, our following research will focus on the dynamic behavior of piezoelectric ceramic actuators under multifield coupling. The error analysis in the article provides a direction for the optimization and improvement of high-speed AFM systems and makes it possible to improve the measurement accuracy further.

## Data Availability

Not applicable.
